# Solitary splenic metastasis of squamous lung cancer: a case report

**DOI:** 10.1186/1757-1626-2-9091

**Published:** 2009-11-25

**Authors:** Diamantis Chloros, Grigorios Bitzikas, Marianna Kakoura, Georgios Chatzikostas, Charlie Makridis, Ioannis Tsitouridis

**Affiliations:** 1 Lung Unit, Papageorgiou Hospital, Ring Road N Efkarpia, Thessaloniki 56403, Greece; 2 Department of Thoracic and Cardiovascular Surgery, Papageorgiou Hospital, Ring Road N Efkarpia, Thessaloniki 56403, Greece; 3 1st Clinic of General Surgery, Papageorgiou Hospital, Ring Road N Efkarpia, Thessaloniki 56403, Greece; 4 Department of Radiology, Papageorgiou Hospital, Ring Road N Efkarpia, Thessaloniki 56403, Greece

## Abstract

**Background:**

Lung cancer is the second commonest malignant tumour, with its splenic metastasis being a rare event.

**Case Presentation:**

We report an exceedingly rare case of a moderate-to-low differentiation squamous cell lung carcinoma in a middle-aged man with a large solitary splenic metastasis detected simultaneously with the primary tumour. Surgical removal of both the primary tumour and the solitary splenic metastasis offered the patient the best treatment option.

**Conclusion:**

The significance of the present case lies on the one hand in the appearance of a large solitary splenic metastasis from a squamous lung cancer at the time of its initial presentation and on the other in the successful excision of both lesions simultaneously.

## Introduction

Lung cancer is the second commonest malignant tumour, with its splenic metastasis being a rare event [1]. When present, it is diagnosed more often at autopsy [2] and it is usually accompanied with metastases to other abdominal organs [1,2]. We report an exceedingly rare case of a squamous cell lung carcinoma in a middle-aged man with a large solitary splenic metastasis detected simultaneously with the primary tumour.

## Case presentation

**Figure 1:**
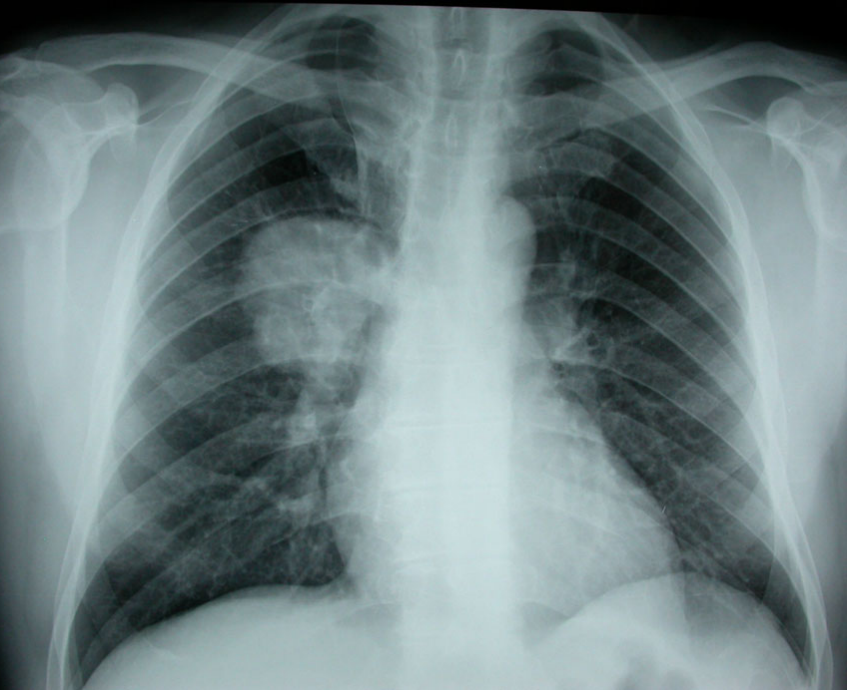
**Plain chest radiograph showing a large homogenous tumour-like opacity in the upper lung zone near the right hilum**.

**Figure 2:**
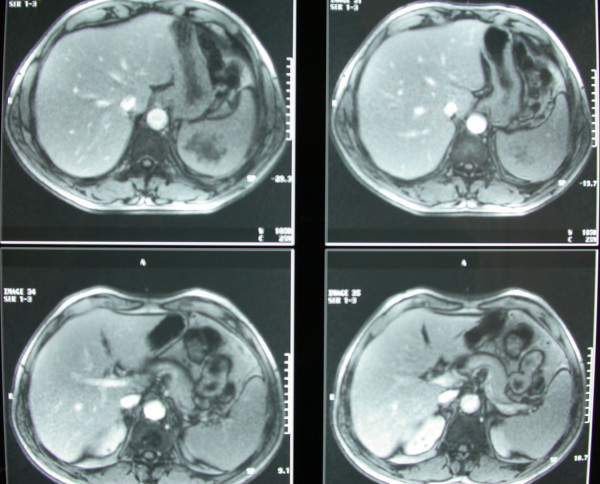
**MRI of the upper abdomen showing a massive solitary lesion in the spleen with a low-density core and a peripheral contrast enhancement suggestive of metastasis**.

A 59-year-old Greek man of Caucasian origin presented with cough and trivial haemoptysis for three days; he was a non-smoker fur-maker in excellent health status, unremarkable medical history and normal physical examination. Chest radiography revealed a homogenous tumour-like opacity (6 cm in diameter) in the upper lung zone near the right hilum (Figure 1). No supraclavicular lymphadenopathy was detected. Computed tomography scanning of the thorax showed a large (6 × 6 cm), almost spherical mass in the right upper lobe and a massive solitary lesion in the spleen (7 cm in its greatest diameter). The mediastinum and the other chest structures were normal. Magnetic Resonance Imaging (MRI) of the upper abdomen showed a massive solitary lesion in the spleen with a low-density core and a peripheral contrast enhancement suggestive of metastasis (Figure 2). Haematological and biochemical test results were normal, except for a slightly elevated level of erythrocyte sedimentation rate (33 mm/h) and of C-reactive protein (2.66 U/L).

Bronchoscopic examination revealed a mass within the posterior segmental bronchus of the right upper lobe and biopsy confirmed the diagnosis of squamous carcinoma of moderate-to-low differentiation. A routine workup was negative for liver, adrenal, brain or bone metastatic lesions. A fine needle biopsy of the spleen under radiological guidance confirmed the diagnosis of metastasis from the primary lung carcinoma. The TNM stage of disease was cT2N0M1 (stage IV). Respiratory function tests were within normal limits.

**Figure 3:**
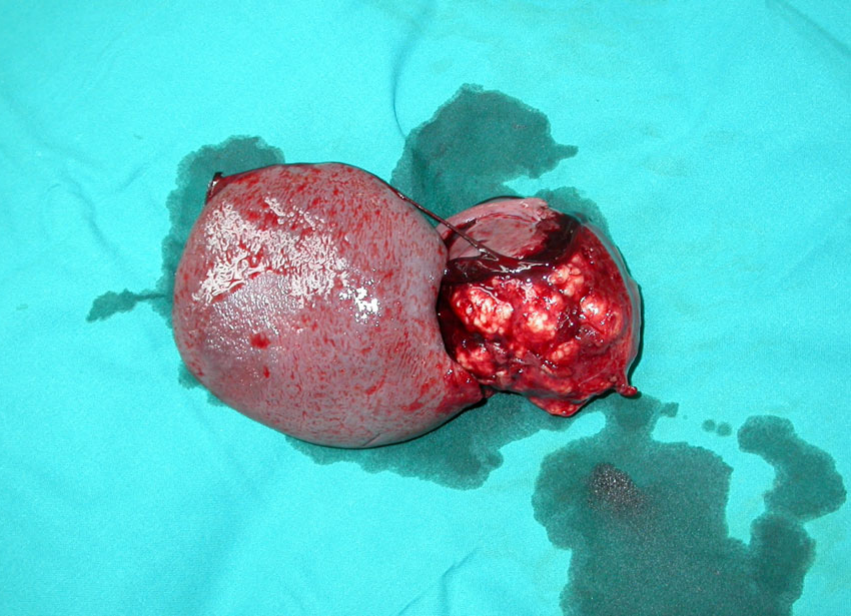
**Gross appearance of the resected spleen with a large (8 cm in diameter) tumour on the upper splenic pole**.

**Figure 4:**
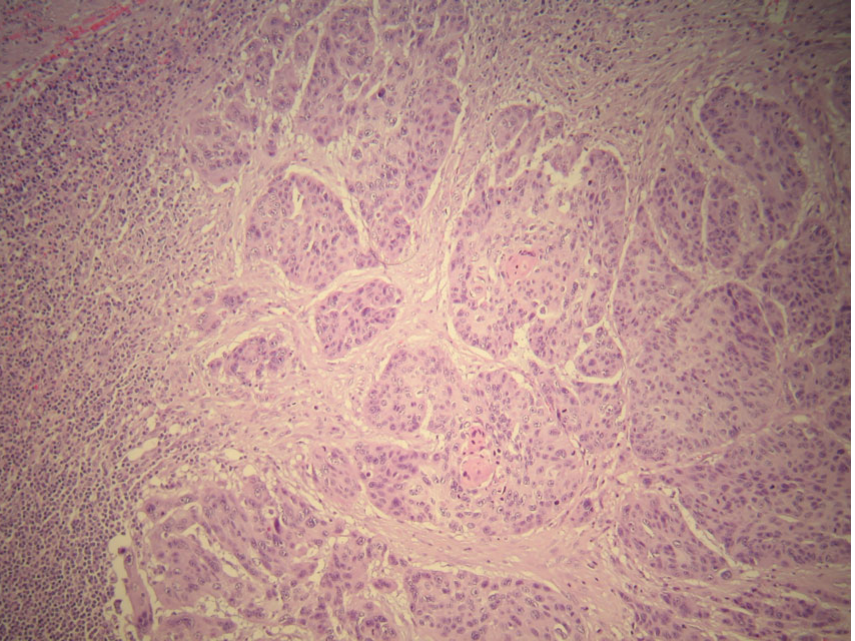
**Microscopic specimen of resected spleen with the characteristic histology of non-small cell lung carcinoma of squamous cell type (Haematoxylin and eosin stain)**.

Surgical removal of both the primary tumour and the solitary splenic metastasis offered the patient the best treatment option. The patient was scheduled for a simultaneous right thoracotomy and splenectomy. Initially a standard right posterolateral thoracotomy was performed. A thorough exploration of the pleural cavity revealed a 6 cm tumor of the right upper lobe that was extended to the apical segment of the lower lobe. Under these circumstances, the intraoperative decision was for a right pneumonectomy, which was finally performed. Five hilar lymph nodes, 2 paratracheal, 2 subcarinal, 2 paraesophageal and 1 from the ligament were excised. After the repositioning of the patient, a typical splenectomy was performed (Figure 3). A microscopic specimen of resected spleen with the characteristic histology of non-small cell lung carcinoma is shown in figure 4. All examined lymph nodes were negative for metastases. The pathological TNM stage of the disease was pT2N0M1. Postoperative course was uneventful and the patient was discharged from the hospital on the 12^th ^postoperative day.

## Discussion

Metastases from lung cancer to the spleen develop usually at advanced cancer stages in the context of disseminated abdominal visceral lesions, while solitary splenic metastasis is extremely rare [3,4]. More unusual is the discovery of a solitary metastasis at the time of the initial lung cancer diagnosis [5,6]. The importance of the present case lies (a) in the appearance of a large solitary splenic metastasis from a moderate-to-low squamous right lung cancer at the time of its initial presentation and (b) in the successful excision of both lesions simultaneously.

Diagnosis can be achieved with splenectomy or with less invasive methods such as fine needle aspiration or transcutaneous biopsy, as in our patient case, with high probability of success and very low complication rate (less than 2%) [7]. The rarity of splenic metastases may be related to the inhibitory effect of the immunologically well-equipped splenic microenvironment on the growth of metastatic cells, given that micro-metastatic dissemination occurs early in the course of malignant disease and is not affected by mechanical factors [3].

Surgical removal of the solitary splenic metastasis is considered the best treatment option. With this aggressive practice, long survival attainment is possible [6] even without adjuvant chemotherapy, which does not appear to be superior to surgery alone [8].

## Consent

Written informed consent was obtained from the next of kin for publication of this case report and accompanying images. A copy of the written consent is available for review by the Editor-in-Chief of this Journal.

## Competing interests

The authors declare that they have no competing interests.

## Authors' contributions

DC and GB were the responsible doctors of the patient. They also analyzed and interpreted the patient data. MK was a major contributor in writing the manuscript. GC and CM performed the surgical operation. IT performed the fine needle biopsy of the spleen. All authors read and approved the final manuscript.

## References

[B1] Satoh H Watanabe K Ishikawa H Yamashita YT Ohtsuka M Sekizawa K Splenic metastasis of lung cancer Oncol Rep 2001 8 1239 1241 11605040 10.3892/or.8.6.1239

[B2] Kinoshita A Nakano M Fukuda M Kasai T Suyama N Inoue K Nakata T Shigematsu K Oka M Hara K Splenic metastasis from lung cancer Neth J Med 1995 47 219 223 8544893 10.1016/0300-2977(95)00011-8

[B3] Comperat E Bardier-Dupas A Camparo P Capron F Charlotte F Splenic metastases: clinicopathologic presentation, differential diagnosis, and pathogenesis Arch Pathol Lab Med 2007 131 965 969 17550328 10.5858/2007-131-965-SMCPDD

[B4] Pramesh CS Prabhudesai SG Parasnis AS Mistry RC Sharma S Isolated splenic metastasis from non small cell lung cancer Ann Thorac Cardiovasc Surg 2004 10 247 248 15458377

[B5] Edelman AS Rotterdam H Solitary splenic metastasis of an adenocarcinoma of the lung Am J Clin Pathol 1990 94 326 328 2396605 10.1093/ajcp/94.3.326

[B6] Scintu F Carta M Frau G Marongiu L Pipia G Casula G Splenic metastases of pulmonary carcinoma. Apropos of a clinical case Minerva Chir 1991 46 1277 1280 1666428

[B7] Keogan MT Freed KS Paulson EK Nelson RC Dodd LG Imaging-guided percutaneous biopsy of focal splenic lesions: update on safety and effectiveness AJR Am J Roentgenol 1999 172 933 937 10587123 10.2214/ajr.172.4.10587123

[B8] Downey RJ Ng KK Kris MG Bains MS Miller VA Heelan R Bilsky M Ginsberg R Rusch VW A phase II trial of chemotherapy and surgery for non-small cell lung cancer patients with a synchronous solitary metastasis Lung Cancer 2002 38 193 197 10.1016/S0169-5002(02)00183-6 12399132

